# Effect of Hydrogen Exposure Temperature on Hydrogen Embrittlement in the Palladium–Copper Alloy System (Copper Content 5–25 wt.%)

**DOI:** 10.3390/ma16010291

**Published:** 2022-12-28

**Authors:** Brandon Roy, Erin LaPointe, Andrew Holmes, Dillon Camarillo, Bonolo Jackson, Daniel Mathew, Andrew Craft

**Affiliations:** Department of Chemistry, University of Hartford, West Hartford, CT 06117, USA

**Keywords:** palladium, copper, hydrogen, embrittlement, exposure temperature

## Abstract

The yield strength, ultimate strength, and elongation/ductility properties of a series of palladium–copper alloys were characterized as a function of the temperature at which each alloy underwent absorption and desorption of hydrogen. The alloys studied ranged in copper content from 5 weight percent copper to 25 wt.% copper. Compared to alloy specimens that had been well-annealed in a vacuum and never exposed to hydrogen, alloys with copper content up to 15 wt.% showed strengthening and loss of ductility due to hydrogen exposure. In these alloys, it was found that the degree of strengthening and loss of ductility was dependent on the hydrogen exposure temperature, though this dependence decreased as the copper content of the alloy increased. For alloys with copper contents greater than 15 wt.%, hydrogen exposure had no discernible effect on the strength and ductility properties compared to the vacuum-annealed alloys, over the entire temperature range studied.

## 1. Introduction

As society transitions away from fossil fuels to alternative energies, the need for pure hydrogen will increase significantly. Along with wind power, solar power, and various other alternative energy sources, hydrogen is expected to play a prominent role in satisfying the world’s energy needs. One limiting aspect of using hydrogen is that pure elemental hydrogen does not exist on the planet. Though abundant, hydrogen is found in nature combined with other elements—for example, the compounds H_2_O and CH_4_. Regardless of the method used, the need to purify hydrogen is a requisite follow-up to every hydrogen production method. Du et al. have given a comprehensive review of the hydrogen purification methods currently used or under development [1]. One of the more common current techniques for purifying hydrogen is the use of hydrogen-specific purification membranes.

The most fundamental criterion that must be satisfied by hydrogen specific membranes is, as the name suggests, they be virtually 100% specific in allowing hydrogen to diffuse through but not any impurities that are in the gas stream. Another important property a membrane must possess is fast transport of hydrogen through the membrane. Palladium membranes possess these two properties and have been thoroughly investigated for possible hydrogen purification utilization [2,3]. In addition to the high financial cost associated with palladium (currently spot priced at over $1800 per ounce), a negative aspect of pure palladium is the significant embrittlement it suffers upon exposure to hydrogen [4,5]. The loss of ductility and the resulting loss of mechanical integrity render pure palladium an inappropriate material for hydrogen purification membranes.

One avenue that is being investigated to alleviate the hydrogen embrittlement problem inherent in pure palladium is to alloy palladium with other metals. The standard bearer at present is a palladium–silver alloy of ~23 wt.% silver [6,7,8]. A major benefit to this alloy versus pure palladium is that the susceptibility to hydrogen embrittlement is significantly reduced [9]. More recently, studies indicate that palladium–copper alloys may be better suited than palladium–silver alloys as effective and efficient hydrogen-specific purification membranes [10,11].

Rigorous investigations attempting to quantitatively characterize the hydrogen embrittlement in palladium–copper alloys recently have been initiated. The study by DiMauro et al. has shown that palladium–copper alloys, of copper content identical to the present study, do enjoy moderately superior resistance to hydrogen embrittlement compared to their palladium–silver counterparts when exposed to hydrogen at 323 K [12]. A preliminary investigation by one of the authors of the present study indicates that resistance to hydrogen embrittlement in palladium–copper alloys extends beyond the compositional limits of the present study and continues to a copper content of at least 50 weight percent [13]. This latter finding is important because the palladium–copper alloy that, at present, has the most optimal properties to function as an effective and efficient hydrogen purification membrane has a copper content of ~53 wt.%.

The present study seeks to build upon the preliminary 323 K results of the earlier investigations by bringing focus to one of the important properties associated with the manifestation of hydrogen embrittlement, namely, the impact of the temperature at which the palladium–copper specimens are exposed to hydrogen. Palladium–copper alloys with copper contents from 5 wt.% to 25 wt.% were studied over a hydrogen-exposure temperature range of 298 K–423 K to characterize the impact of hydrogen exposure temperature on strength and ductility, and the results are presented here.

## 2. Materials and Methods

Palladium–copper (99.9% pure metal basis) foils (ACI Alloys, San Jose, CA, USA) of 0.25 mm thickness were used in this study. These foils were unidirectionally cut into 38.1-mm-long by 6.4-mm-wide specimens. A reduced section of 3.2 mm width was machined into each specimen to facilitate a break in the middle during tensile testing. Specimens were lightly abraded with fine emery paper and then chemically polished in a 2:2:1 volume mixture of H_2_SO_4_:HNO_3_:H_2_O, followed by liberal rinsing, in an ultrasonic cleaner, with deionized water and then acetone. All specimens were then stress relieved in vacuo at 723 K for 48 h, followed by a 24 h annealing in vacuo at 823 K. These annealing temperatures were high enough to allow recovery of each specimen to a nearly defect-free state. Some of the vacuum-annealed specimens were retained for strength testing to establish baseline values against which hydrogen-treated specimens would be compared.

Each vacuum-annealed specimen subjected to hydrogen exposure experienced a single isothermal hydrogen absorption/desorption cycle as follows. Hydrogen absorption/desorption was carried out in an all-stainless-steel system of calibrated volumes connected to a commercial tank of ultrapure hydrogen. The temperature of the specimen chamber of the system was maintained by a regulated water bath for temperatures below 373 K and by a computer-controlled furnace for temperatures at and above 373 K. Hydrogen gas pressures were measured with MKS diaphragm gauges. Specimens were exposed to a hydrogen pressure of 1.33 × 10^5^ Pa and allowed to absorb hydrogen until no further hydrogen pressure decrease was observed, indicating that hydrogen absorption had ceased. Upon completion of hydrogen absorption, specimens were evacuated for 24 h at the absorption temperature to remove all absorbed hydrogen. The hydrogen desorption was carried out via a vacuum system composed of a HyVac two-stage pump (HyVac Products, Pottstown, PA, USA). Complete absorption/desorption cycling was carried out isothermally on respective specimens at 25 K increments from 298 K to 423 K. It is important to note that, although different cycling temperatures distinguish different sets of specimens for each alloy studied, each cycling treatment on a specific set of specimens was carried out at constant temperature.

Tensile tests were carried out on both vacuum-annealed and hydrogen-cycled specimens using an Instron Series IX Automated Materials Testing System (Instron Corporation, Norwood, MA, USA) using a gauge length of 19 mm and a constant elongation rate of 1.27 mm/min. Three specimens corresponding to each respective treatment (vacuum-annealed and hydrogen-cycled) underwent tensile testing.

## 3. Results and Discussion

**Figure 1:**
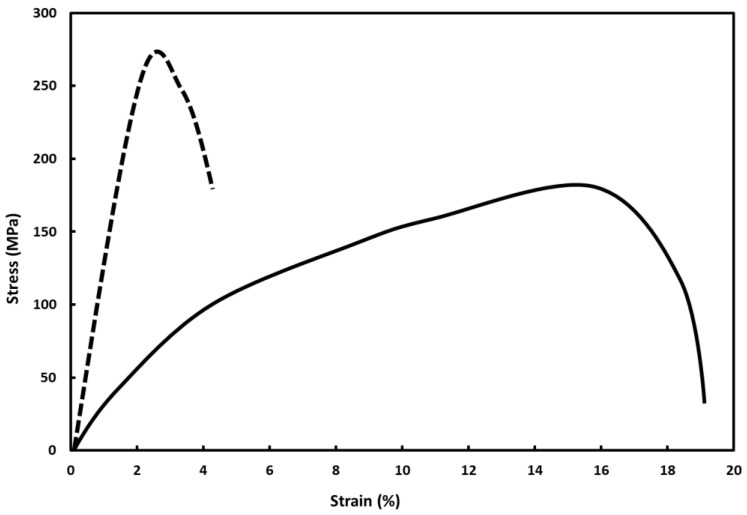
Representative engineering stress–strain curves for vacuum-annealed (solid curve) and hydrogen-cycled at 298 K (dashed curve) palladium–copper (5 wt.% copper) alloy.

Figure 1 shows representative engineering stress–strain curves that were part of the current investigation, in particular, the stress–strain curves for vacuum-annealed palladium–copper (5 wt.% copper) and the palladium–copper (5 wt.% copper) alloy hydrogen cycled at 298 K.

Analysis of the tensile stress–strain curves from this study allowed the determination of the yield strength, ultimate strength, and elongation at failure for each alloy investigated.

Table 1 reports the yield strength, ultimate strength and elongation at failure for the vacuum-annealed alloys studied. The values for pure palladium are included for comparison purposes.

The results for the vacuum-annealed alloys are consistent with those obtained in the earlier study of DiMauro et al. [12]. As the values in Table 1 clearly show, the palladium–copper alloy system manifests solid-solution strengthening over the composition range studied. Though interesting in their own right, these results for the mechanical properties of the vacuum-annealed palladium–copper alloys serve as the baseline values against which the values for alloys exposed to hydrogen will be compared to discern any hydrogen embrittlement that results from the hydrogen absorption/desorption treatments. Investigations on pure palladium [5] and palladium–silver alloys [9] have shown that hydrogen embrittlement results in the strengthening of the metal and loss of ductility, compared to vacuum-annealed alloys that have not been exposed to hydrogen. Therefore, the tell-tale signs of hydrogen embrittlement are well established and should be straightforward to discern in the palladium–copper alloys of the current study.

**Figure 2:**
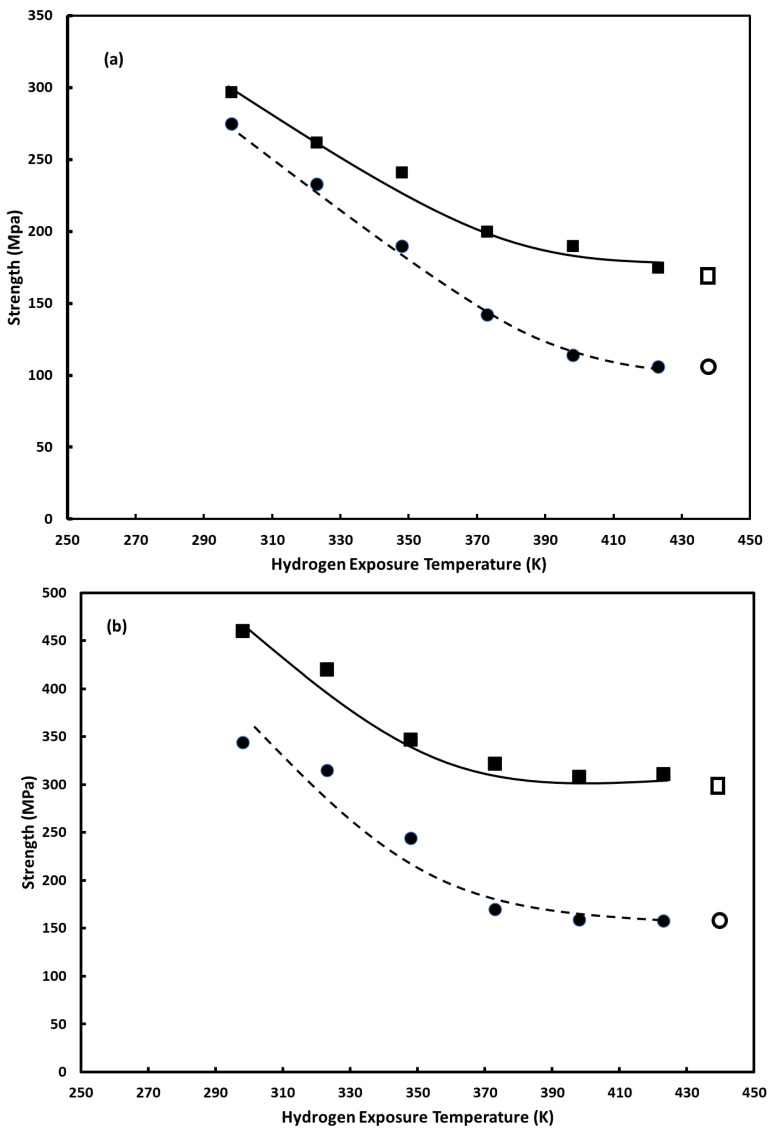

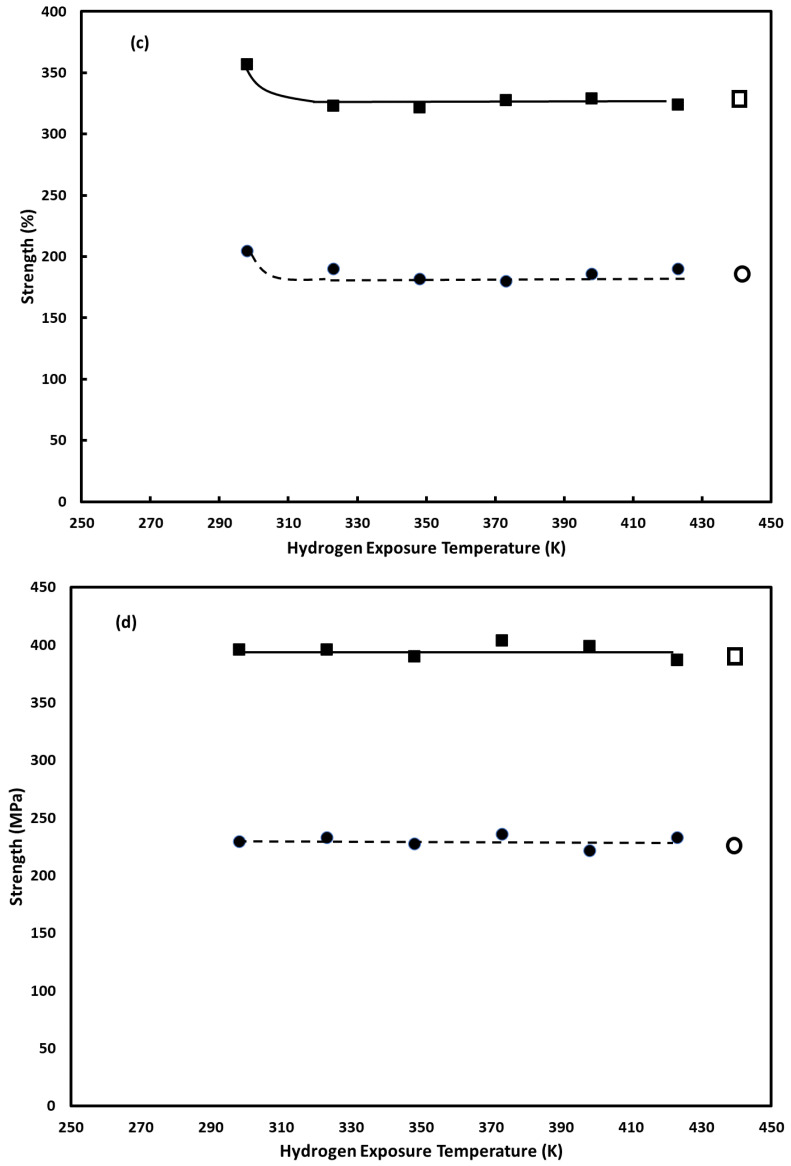

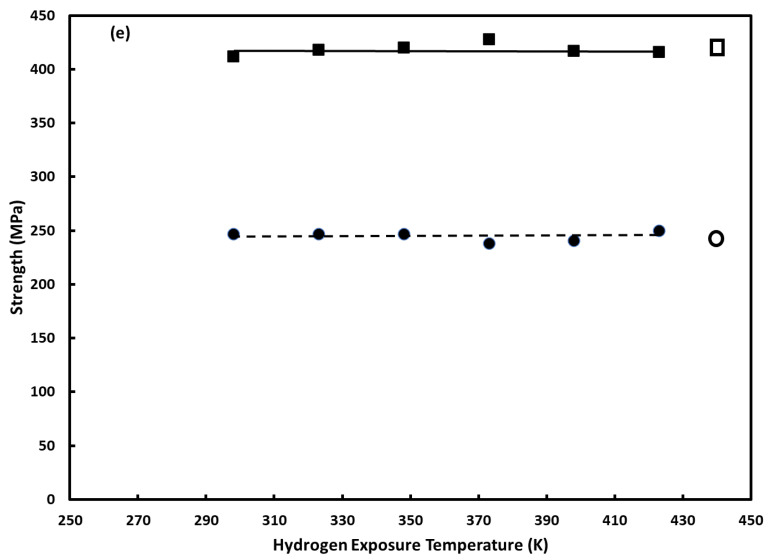
Yield Strength (●) and ultimate strength (■) as a function of hydrogen absorption/desorption cycling temperature: (**a**) palladium–copper (5 wt.% Cu); (**b**) palladium–copper (10 wt.% Cu); (**c**) palladium–copper (15 wt.% Cu); (**d**) palladium–copper (20 wt.% Cu); (**e**) palladium–copper (25 wt.% Cu). For comparison, yield strength (○) and ultimate strength (□) of vacuum-annealed specimens are included and positioned at an arbitrary temperature.

**Figure 3:**
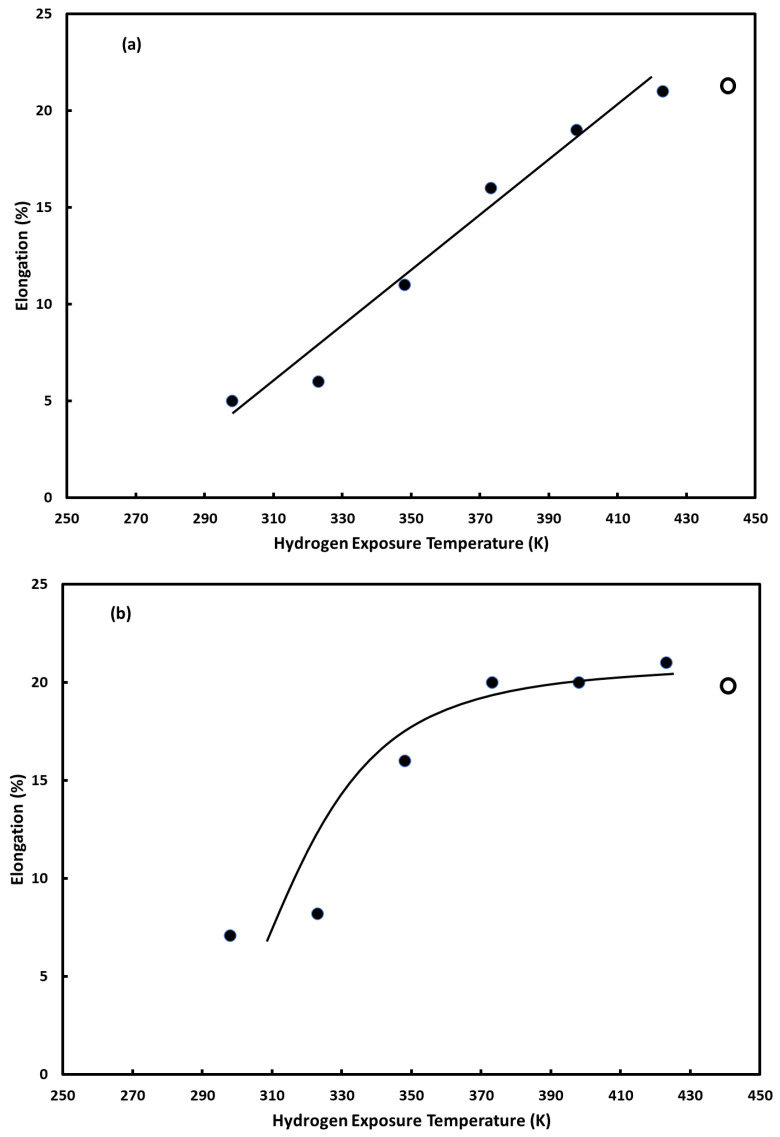

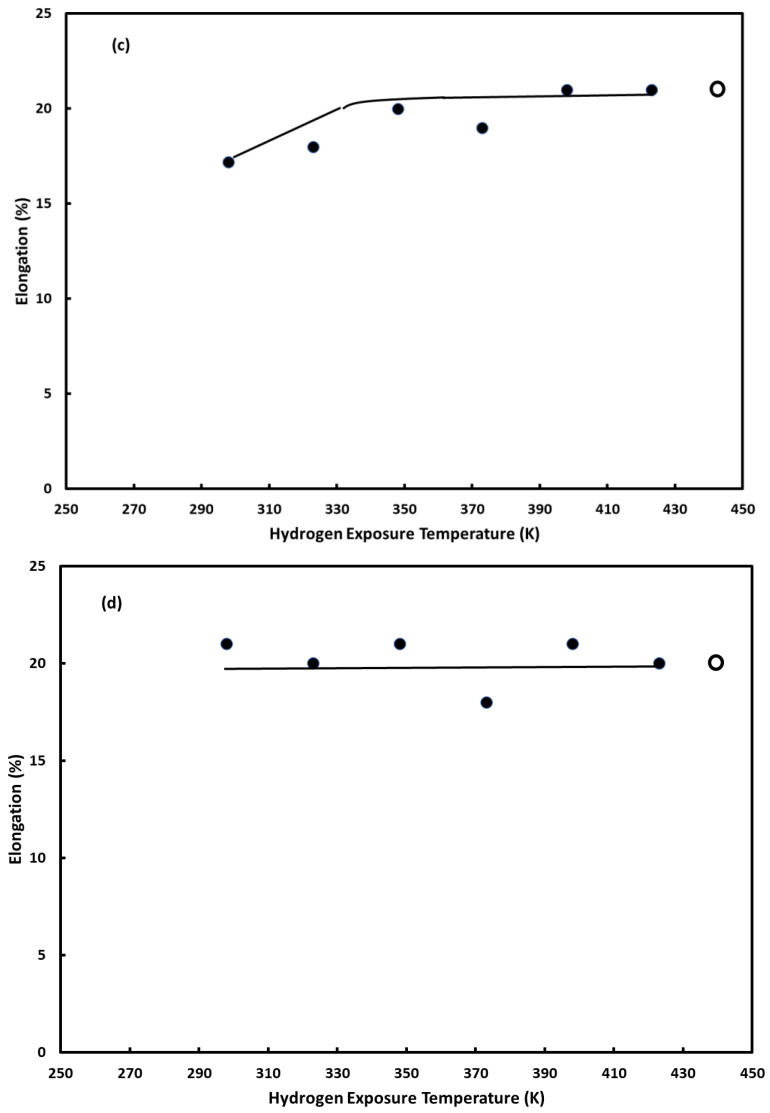

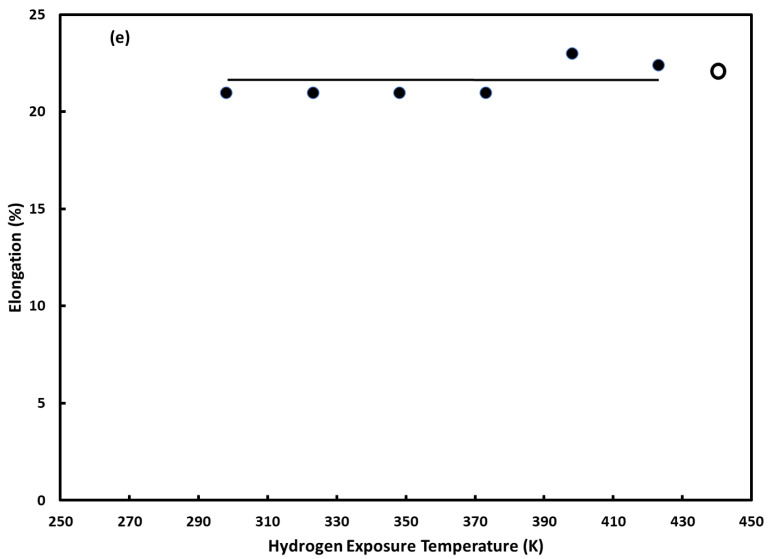
Elongation at failure as a function of hydrogen absorption/desorption cycling temperature: (**a**) palladium–copper (5 wt.% Cu); (**b**) palladium–copper (10 wt.% Cu); (**c**) palladium–copper (15 wt.% Cu); (**d**) palladium–copper (20 wt.% Cu); (**e**) palladium–copper (25 wt.% Cu). For comparison, elongation at failure (○) of vacuum-annealed specimens is included and positioned at an arbitrary temperature.

Figure 2 shows the plots of yield strength and ultimate strength as a function of hydrogen exposure temperature for the alloys studied. In each plot, the value for the vacuum-annealed form of each alloy is included at an arbitrary location as reference. Figure 3 shows the plots of total elongation (i.e., elongation at failure) as a function of hydrogen exposure temperature for the alloys studied. As with Figure 2, in each plot, the value for the vacuum-annealed form of each alloy is included as reference. In each figure, plot a is for the 5 wt.% Cu alloy; plot b for the 10 wt.% Cu alloy, plot c for the 15 wt.% Cu alloy; plot d for the 20 wt.% Cu alloy; plot e for the 25 wt.% Cu alloy. As can be seen in the figures, the copper content of the alloy plays a prominent role in the sensitivity of the measured properties to hydrogen exposure temperature. Recall, the greater the difference in a mechanical property for a hydrogen-cycled specimen from the corresponding value for a vacuum-annealed specimen, the greater the hydrogen embrittlement.

The results for the palladium–copper (5 wt.%) alloy show a marked dependence of all three measured properties on the hydrogen exposure temperature over the majority of the temperature range studied (298–423 K). The exception is for the alloy exposed to hydrogen at 423 K, whose values appear to be in line with those of the vacuum-annealed alloy. As the results in Figure 2a show, with the exception of the alloy exposed to hydrogen at 423 K, both the yield strength and ultimate strength are enhanced by exposure to hydrogen, compared to the vacuum-annealed alloy. The degree of strengthening created by the absorption/desorption of hydrogen is clearly delineated in the plot, with the degree of strengthening decreasing as the hydrogen exposure temperature increases. As shown in Figure 3a, the elongation at failure of the 5 wt.% copper alloy also depends on the hydrogen exposure temperature. The loss of ductility is the most telling sign that the detrimental effects of hydrogen embrittlement have occurred. Like the strengthening caused by the hydrogen exposure treatments, the loss of ductility is dependent on the hydrogen exposure temperature. The lower the hydrogen exposure temperature, the greater the loss of ductility. Like the strength results, the 5 wt.% copper alloy exposed to hydrogen at 423 K did not experience any perceptible loss of ductility, compared to the vacuum-annealed 5 wt.% alloy. Therefore, in the palladium–copper (5 wt.% Cu) alloy, strength and ductility evidence indicate that hydrogen embrittlement, to varying degrees, occurs up to a hydrogen-exposure temperature of at least 398 K, and is no longer observed at a hydrogen-exposure temperature of 423 K.

A similar analysis of the results for the palladium–copper (10 wt.%) alloy shows evidence of hydrogen embrittlement but over a more limited range of hydrogen exposure temperatures. In particular, the strengthening and loss of ductility indicative of hydrogen embrittlement is evident up to a hydrogen exposure temperature of ~373 K. At temperatures above 373 K, the strength and elongation properties are in line with those of the vacuum-annealed alloys.

The decrease in the temperature range over which hydrogen embrittlement manifests as copper content increases continues with the remaining alloys. For the 15 wt.% alloy, the strengthening and loss of ductility appear to manifest, albeit modestly, up to a temperature of ~323 K. The results for the remaining palladium–copper alloys show that hydrogen exposure over the entirety of the temperature range studied has no perceptible effects on their strength and ductility properties, compared to the vacuum-annealed alloys.

The results clearly show that the copper content of the alloys plays a vital role in the ability of palladium–copper alloys to resist the onset of hydrogen embrittlement. For applications that involve interactions with hydrogen at temperatures above 298 K, alloys with copper contents greater than ~15 wt. percent will not suffer the deleterious effects of hydrogen embrittlement.

The present results lend support to the explanation offered by DiMauro et al. [12]. Those authors explained their observed variations, as a function of copper content, in the strength, hardness, and elongation of palladium–copper alloys exposed to hydrogen at 298 K as resulting from copper’s effect on the miscibility gap of the metal-hydrogen system.

**Figure 4:**
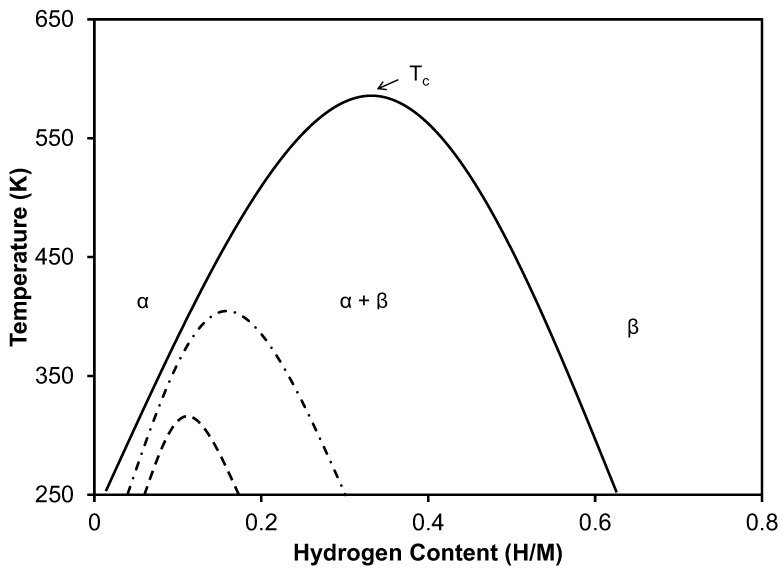
Schematic temperature-composition phase diagram for the palladium–hydrogen system (solid line) and palladium-based alloy-hydrogen systems (dashed lines).

Hydrogen embrittlement in the palladium hydrogen system has been ascribed primarily to the dislocations generated during hydrogen absorption and desorption due to the discontinuous phase change from a dilute face-centered cubic solid solution of hydrogen in the palladium matrix to a dense face-centered cubic solid solution of hydrogen in the palladium matrix, referred to as a hydride phase. The discontinuous nature of the phase change in the Pd-H system is best appreciated by referencing the temperature-composition phase diagram, a schematic of which is shown in Figure 4. The dilute solid solution is referred to in the figure as the α phase while the dense hydride phase is referred to as the β phase. At temperatures that cause the palladium matrix to pass through the (α + β) coexistence miscibility gap during hydrogen absorption/desorption, dislocations will be generated due to the abrupt discontinuous volume change experienced by the metal matrix. For example, at 298 K, the β hydride phase is ~10% larger than the dilute α solid solution [14]. As the phase diagram indicates, as temperature increases, the width of the miscibility gap decreases, eventually collapsing into the critical point at ~570 K. As the width of the miscibility gap decreases, the volume difference between the α and β phases decreases, with a concomitant decrease in the number of dislocations generated during the α → β phase change (during hydrogen absorption) and the β → α phase change (during hydrogen desorption). At temperatures above the critical temperature, the α → β and β → α phase changes occur in a continuous homogeneous manner with very few dislocations generated by the phase change.

The alloys involved in the current study reside in the region of the palladium–copper binary system exclusively comprised of homogeneous solid solutions exhibiting a face-centered cubic structure [15]. In this regard, the palladium–copper system is similar to the better-studied palladium–silver system. Much like the effect of alloying palladium with silver, the alloying of palladium with copper has been found to depress the critical temperature of the miscibility gap and decrease the width of the miscibility gap [16,17,18,19]. The degree to which the miscibility gap is depressed, and width decreased is directly proportional to the copper content of the alloy. This is illustrated generically in Figure 4.

The present strength and ductility results are consistent with a depression of the critical temperature and a decrease in the width of the miscibility gap in the palladium–copper–hydrogen system as the copper content increases. The results imply that the critical point for the 5 wt.% Cu alloy is ~423 K, while that for the 10 wt.% Cu alloys is ~373 K, and that for the 15 wt.% Cu alloy is ~323 K. For the 20 and 25 wt.% Cu alloys, the present results indicate that the critical temperature of the miscibility gap is depressed to a temperature below 298 K (the lowest temperature studied in the current investigation). However, further studies are warranted to see if other factors contribute to the observed changes in the strength and ductility found in the present study. It is known that hydrogen exposure can generate vacancies [20] in a metallic lattice and changes to grain and sub-grain fine structures of metals [21]. These changes may contribute to the changes in mechanical properties observed in palladium–copper alloys.

## 4. Conclusions

The present results indicate that the temperature at which palladium–copper alloys of copper weight percent ≤25 are exposed to hydrogen can have a significant effect on the degree of hydrogen embrittlement experienced by the material. As the weight percent copper increases, the alloy becomes more resistant to the damaging effects of hydrogen embrittlement, with a copper content in excess of 15 weight percent copper showing no signs of hydrogen embrittlement over the entire temperature range studied. The present results lend support to the importance of the miscibility gap in a metal-hydrogen system and its role in contributing to hydrogen embrittlement. In order to avoid the ravages of hydrogen embrittlement, the present results indicate that the material should avoid conditions that cause the metal-hydrogen system to traverse the miscibility gap.

## Figures and Tables

**Table 1:** Yield strength, ultimate strength, and total elongation for vacuum-annealed Palladium and Palladium–Copper Alloys.

Title	Vacuum-Annealed Pd	Vacuum-Annealed Pd/Cu (5%)	Vacuum-Annealed Pd/Cu (10%)	Vacuum-Annealed Pd/Cu (15%)	Vacuum-Annealed Pd/Cu (20%)	Vacuum-Annealed Pd/Cu (25%)
Yield strength (MPa)	63	108	156	182	229	247
Ultimate strength (MPa)	146	170	302	323	396	418
Total Elongation (%)	20	20	19	21	20	22

## Data Availability

Data are available upon request from the corresponding author.
